# The Human Otubain2-Ubiquitin Structure Provides Insights into the Cleavage Specificity of Poly-Ubiquitin-Linkages

**DOI:** 10.1371/journal.pone.0115344

**Published:** 2015-01-15

**Authors:** Mikael Altun, Thomas S. Walter, Holger B. Kramer, Patrick Herr, Alexander Iphöfer, Johan Boström, Yael David, Alia Komsany, Nicola Ternette, Ami Navon, David I. Stuart, Jingshan Ren, Benedikt M. Kessler

**Affiliations:** 1 Target Discovery Institute, Nuffield Department of Medicine, Roosevelt Drive, University of Oxford, Oxford, OX3 7FZ, United Kingdom; 2 Division of Structural Biology, Nuffield Department of Medicine, Roosevelt Drive, University of Oxford, Oxford, OX3 7BN, United Kingdom; 3 Department of Physiology, Anatomy and Genetics, South Parks Road, Oxford, OX1 3DQ, United Kingdom; 4 Science for Life Laboratory, Division of Translational Medicine and Chemical Biology, Department of Medical Biochemistry and Biophysics, Karolinska Institutet, SE-171 21 Stockholm, Sweden; 5 Helmholtz Centre for Infection Research, Inhoffenstrasse 7, 38124 Braunschweig, Germany; 6 Department of Biological Regulation, The Weizmann Institute of Science, Rehovot 76100, Israel; University of Alabama at Birmingham, UNITED STATES

## Abstract

Ovarian tumor domain containing proteases cleave ubiquitin (Ub) and ubiquitin-like polypeptides from proteins. Here we report the crystal structure of human otubain 2 (OTUB2) in complex with a ubiquitin-based covalent inhibitor, Ub-Br2. The ubiquitin binding mode is oriented differently to how viral otubains (vOTUs) bind ubiquitin/ISG15, and more similar to yeast and mammalian OTUs. In contrast to OTUB1 which has exclusive specificity towards Lys48 poly-ubiquitin chains, OTUB2 cleaves different poly-Ub linked chains. N-terminal tail swapping experiments between OTUB1 and OTUB2 revealed how the N-terminal structural motifs in OTUB1 contribute to modulating enzyme activity and Ub-chain selectivity, a trait not observed in OTUB2, supporting the notion that OTUB2 may affect a different spectrum of substrates in Ub-dependent pathways.

## Introduction

Modification by ubiquitin and other members of the ubiquitin family (ubiquitin-like proteins, Ubls), plays a key role in controlling the fate, lifespan, localization and function of the majority of proteins in eukaryotic cells [1]. The ubiquitin or Ubl C-terminal tail is covalently attached to protein lysyl side chains via an isopeptide bond, a process controlled by ubiquitin or Ubl conjugating enzymes [2]. This modification is reversed by deubiquitylating enzymes (DUBs) or Ubl-specific proteases [3], rendering this a dynamic process the importance of which is underscored by the capacity of hundreds of enzymes to control this reaction. DUBs or Ubl-specific proteases are catalytic cysteine- or metallo-proteases that contain Ubiquitin/Ubl recognition motifs. Amongst them is a family of proteases sharing a conserved domain, the ovarian tumor domain (OTU) containing a cysteine protease motif [4].

Otubain-1 and Otubain-2 were the first two OTU proteins found to display *in vitro* DUB activity [5, 6]. OTUB1 appears to modulate levels of proteins involved in immune regulation [7–9] and cancer through catalytic activity-dependent and independent mechanisms [10–12]. A peculiarity of OTUB1 is its ability to inhibit ubiquitination by binding to E2~Ub thiolesters, such as UBC13, and prevent the transfer of Ub to E3 Ubiquitin ligases [13, 14]. In turn, E2 binding to OTUB1 also controls its DUB enzyme activity [15]. Interestingly, OTUB2 has recently been described to fine-tune DNA damage-dependent ubiquitination and thereby influence the choice of DNA repair pathways [16]. It may also contribute to Tumor necrosis factor associated factors 3 and 6 (TRAF3/6) turnover and is expressed at highest levels in the brain [5, 7].

The recently determined structure of the human OTUB2 apo enzyme shows that, unlike other cysteine protease DUBs such as OTUD1, OTUD2 and OTUD3, the catalytic triad is stabilized in a functionally incompetent form by a unique hydrogen bonding network configuration [17], confirming that there are clearly distinct functional subclasses within the OTU family [18]. Another catalytically incompetent conformation is observed for the OTUB1 apo structure [19] that rearranges when OTUB1 is in complex with Ub and UBC13 [13, 14], also observed in the related yeast ovarian tumor 1 (yOTU1) domain in complex with Ub [20]. Structural information has also begun to illuminate the specificity of OTUs towards other Ubls. For instance, vOTUs also process Interferon stimulated gene 15 (ISG15) to modulate the host antiviral response, a trait not readily observed for mammalian OTUs, due to a different ligand binding mode [21–24]. In addition, co-crystal structures of OTUB1 in complex with UBC13 and Ub molecules revealed more details on the molecular recognition of different Ub-chain linkages, demonstrating a predominant role of the proximal Ub in determining Ub-linkage specificity [13, 14], consistent with biochemical studies on a panel of the OTU protein family [18]. To further understand aspects of the molecular basis of discriminating between different Ub chain linkages and Ubls by OTUs, we set out to co-crystallize human OTUB2 covalently bound to ubiquitin through the reaction with ubiquitin 2-bromoethyl (Ub-Br2). Functional comparison with OTUB1 revealed a role for the N-terminal domain in modulating enzymatic cleavage.

## Materials and Methods

Cloning, expression and purification of OTUB2 and the generation of HA-tagged ubiquitin 2-bromoethyl (HA-Ub-Br2) probe were performed as described previously [19]. In order to obtain the OTUB2-HA-Ub complex, ~6mg recombinant OTUB2 was incubated with aequimolar (~2mg) HA-Ub-Br2 for 120 min at 37°C, followed by purification over gel filtration using a Sephadex 200 16/60 column in 20mM HEPES pH 8.0, 50mM NaCl, 0.5mM TCEP buffer on an Akta FPLC system. Recombinant OTUB1 and OTUB2 were prepared as reported previously [19]. Recombinant UCH-L3 was generously provided by Dr. Benjamin Nicholson (Progenra Inc.). The generation, expression and purification of additional recombinant DUBs used in this study are described in the Supporting Information section.

### Protein crystallization

The purified complex of OTUB2-HAUb was concentrated to 16 mg/mL using a centrifugal concentrator (10 000 MWCO, Vivascience) and deemed to be appropriate for crystallization trials as judged by a Pre-Crystallization Test (PCT, Hampton Research, CA, USA). As described in [25], primary screening experiments, set up as 100 nL + 100 nL sitting drops with a Cartesian HoneyBee X8 instrument (Digilab, Huntingdon, UK) and equilibrated against a reservoir of 95 µL, were monitored at both 6 °C and 21 °C with imaging systems (Veeco/TAP and Formulatrix), respectively. A cluster of small rods grown from a single nucleation centre were observed after 12 days in 15% (w/v) Polyethylene Glycol 3350, 0.1 M Magnesium Formate, at 6 °C, and continued to grow for a further week. Single rod-like crystals could be separated from the clusters and were collected for analysis.

### Data collection and structure determination

X-ray data were collected at beam line I04–1, Diamond Light source using a Pilatus 2M detectors from 2 crystals at a wavelength of 0.9173 Å. A total of 1800 frames, 0.2° each, were collected to give a data set that has 99.1% completeness and a redundancy of 9.0 to 2.05 Å resolution. X-ray data indexing, integration and scaling were done using HKL2000 [26]. Molecular replacement solution was obtained with MOLREP [27] using searching models of apo OTUB2 (PDB ID: 1TFF) [17] and Ub (PDB ID: 3N32). Cyclic model rebuilding with COOT [28] and refinement with PHENIX [29] have resulted in the current structure. Data collection and refinement statistics are shown in S1 Table Structural comparisons used SHP [30]. Structure figures were prepared using PYMOL [31]. The PDB ID of the deposited structure is 4FJV.

### Ub/Ubl isopeptidase assays using linear di-ubiquitin, di- ubiquitin, Lys48-/Lys63-linked tetra-ubiquitin and di-SUMO

Linear di-ubiquitin, tetra-ubiquitin (Lys48 and Lys63), di-SUMO and UB/Ubl substrate isopeptidase assays were performed essentially as described previously [19]. In brief, poly-linked (BIOMOL), di-linked (Boston Biochem) Ub and HA-Ub-probe assays were performed with 1 µM of the recombinant DUB enzyme(s), 10 µM di-Ub, 100ng of poly-linked Ub chains (BIOMOL) and 1 µg of HA-Ub-probes for 4 hours at 37°C in 50mM tris (pH 8.0) and 1mM DTT. Reactions were terminated with 3x reducing sample buffer and proteins separated by SDS-PAGE and visualized by immunoblotting with an anti-Ub (BD Pharmingen; 1:3,000) or anti-HA-HRP (Sigma; 1:10,000) antibody.

### Production of Ub/Ubl substrates and TR-FRET-ubiquitin

The biotinylated peptide isopeptide assay substrate was prepared as previously described [19, 32]. Fluorescein-ubiquitin and LanthaScreen Thiol Reactive Tb Chelate were purchased from Invitrogen (UK), and ubiquitin-AMC (Ub-AMC) from Boston Biochem (Cambridge, MA, USA). The Ub-AMC assay and the protocol for conjugating peptide to Ub/Ubl was performed as described above.

To perform a ubiquitin protein-based isopeptidase assay that better reflects the cleavage specificity of DUBs, we developed a time-resolved fluorescence resonance energy transfer (FRET)-based isopeptide DUB substrate. Our strategy as described below was to conjugate a fluorescence group/ubiquitin-peptide instead of a biotinylated peptide to the C-terminus of ubiquitin via an isopeptide bond. To this end, a peptide sequence including Ub Lys27/Lys29 containing N-terminal cysteine (CVKAKIQD) was used. The cysteine group of the peptide was labeled via its reaction with a maleimide moiety of the thiol-reactive Tb chelate (Invitrogen, UK). DTT and excess unconjugated peptide were removed by concentrating the reaction mixture four times with 50 mM TRIS pH 7.8 using centrifuge concentrators Vivaspin (Vivaspin, 5,000 MWCO, Sartorius Stedim Biotech S.A., and Aubagne Cedex, France). The Tb-maleimide labeling reaction was started by adding Tb chelate (190 µM) and incubated for 12 h at room temperature in the dark. The product was then washed twice with Vivaspin (30 000 MWCO), concentrated 2x with Vivaspin (5,000 MWCO) and stored at 20°C. Measurements using the TR-FRET-Ubiquitin are described below.

### TR-FRET-ubiquitin cleavage assays

50 nM of the fluorescein-ubiquitin-isopeptide TR-FRET DUB substrate was incubated with 50nM recombinant OTUB1, OTUB1 P87G, OTUB2 or 1.25 nM UCH-L3 in a final volume of 100 µl in with Corning 96 well plates. Cleavage was measured as a ratio function of acceptor fluorescence to donor fluorescence (515/487 nm emission) as a function of time by 332 nm excitation on the Tecan Safire² Monochromator Based Plate Reader with 20 nm band pass. The substrate construct shows TR-FRET between terbium and fluorescein, and DUB-dependent cleavage leads to a decrease in FRET signal. Because of the expensive thiol reactive terbium chelate the improvement of the signal was omitted. However, this approach shows a suitable functional TR-FRET principle. A significant advantage of the TR-FRET format is the time-resolved and ratio metric nature of this assay, and problems typically resulting from autofluorescent compounds, precipitated compounds, or colored compounds are thus generally eliminated.

### Ubiquitin-AMC (7-amino-4-methylcoumarin) based assays

Ubiquitin-AMC assays were performed essentially as described previously [19].

### Cloning, expression and purification of OTUB1-OTUB2 chimeric and OTUB mutant proteins

Recombinant OTUB2ΔC5 (lacking the C-terminal residues 229–234 and catalytically inactive mutant OTUB2ΔC5 C51S used in S1 Fig. were prepared as reported previously [5]. The cDNA representing OTUB1–2 and OTUB2–1 chimeric sequences (S2 Fig.) was synthesized by GeneArt (Germany) and subsequently cloned into pET28alpha vector (BamHI and HindIII) for bacterial expression and pCMV10 for mammalian expression (NotI and KpnI). The constructs were verified by DNA sequencing. Expression and purification of the OTUB1-OTUB2 chimeric proteins (OTUB1–2, OTUB2–1) was performed as described previously for human OTUB1 and OTUB2 [19]. The recombinant proteins were examined for their molecular weight by mass spectrometry analysis: OTUB1–2: observed [M+H]^+^ 35,095 (minus N-terminal Met), calculated mass 35,094 Da (minus N-terminal Met); OTUB2–1: observed [M+H]^+^ 30,233 (minus N-terminal Met), calculated mass 30,232Da (minus N-terminal Met). The recombinant OTUB1 and OTUB2 proteins were [M+H]^+^ 33,176 (calculated mass 33,179 Da) and 29,534 (calculated mass 29,534 Da), all masses without N-terminal Met, respectively (see also [19].

## Results and Discussion

### Structure of human OTUB2 bound to Ub

**Figure 1:**
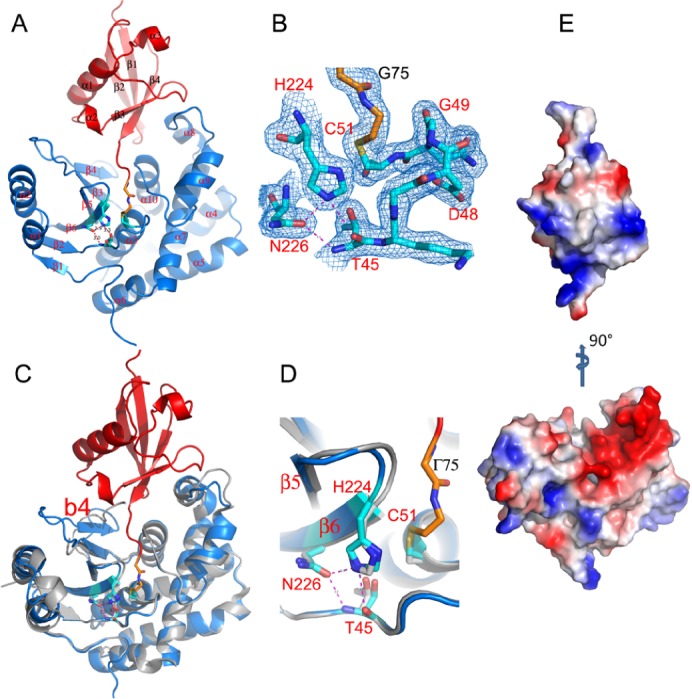
Overall structure of the OTUB2-Ub complex. (**A**) A ribbon diagram showing OTUB2-Ub complex with OTUB2 colored in blue and Ub in red. The active site residues are shown as sticks with carbon atoms colored in cyan; the last two residues of Ub and the covalent linker to Cys51 are drawn as orange sticks. (**B**) Representative *|2Fo-Fc|* map contoured at 1σ showing well defined electron density for residues around the active site. (**C**) Comparison of ligand bound and apo (grey) structures of OTUB2, the large structural changes due to Ub binding are indicated by arrows. (**D**) Close-up view of the active site of ligand bound and apo OTUB2. (**E**) Electrostatic surfaces of OTUB2 (bottom panel) and Ub (top panel). Ub is moved upwards and rotated 90° to show the positively charged surface patch that has complementary interactions with OTUB2.

A protein complex of human OTUB2 and Ub, an active site directed probe, was obtained by the irreversible formation of the covalent thioether bond between the catalytic site cysteine 51 of OTUB2 and the ethane bromoethyl moiety at the C-terminus of Ub. Protein complexes purified to homogeneity yielded crystals which diffracted to a resolution of 2.05 Å. The structure was determined by molecular replacement method and refined against all the x-ray data to an R-factor 0.212 (R-free 0.269) with rsmd’s of 0.010 Å for bond lengths and 1.2° for bond angles from ideal values (S1 Table). The final refined model contains residues 2–232 of OTUB2 and 1–75 of Ub in each OTUB2-Ub complex (Fig. 1). In addition, six residues from the N-terminal region of each Ub are ordered and interact with symmetry related molecules. OTUB2 is comprised of a 6-stranded β-sheet sandwiched by two helical domains. The overall structure of the OTUB2-Ub complex shows an Ub binding mode similar to other DUBs in that the Ub C-terminal tail is extended and reaches to the catalytic centre (Fig. 1A). The covalent thioether bond formed between Ub-Gly75-bromo ethyl and OTUB2 Cys51 (Fig. 1B), and the catalytic residues Cys51, His224, Asn226 and Thr45 are all well defined in the electron density map and within sufficient proximity to engage in a hydrogen bonding network. A comparison of the OTUB2 apo [17] and Ub ligand bound structures illustrates only subtle movements of these residues within the catalytic centre (Fig. 1C and D). However, large conformational changes and local re-structuring occur at both sides of the Ub binding cleft of OTUB2 upon Ub binding. Residues 197–204 are disordered and 205–208 fold into the Ub binding cleft in the apo OTUB2 structure. In the complex, residues197–204 become ordered and 205–208 form a β-strand (β4) antiparallel to β3 with a movement of 12.6 Å for the Cα atom of residue 205. The newly ordered β3β4 loop packs against the inner face of α2 helix and introduces a subtle reorientation of the α1 and α2 helices and conformational changes of α1α2 loop and β2 strand. On the other side of the cleft there is a 5 Å sideways movement of α8α9 loop. 201 Cα atoms out of 232 liganded OTUB2 can be overlapped with the apo enzyme with an rmsd of 0.8 Å (Fig. 1C).

### The OTUB2-Ub interface

The OTUB2-Ub interaction surface is characterized by a large positively charged surface patch contributed by Arg42, Lys48 and Arg72 that has complementary interactions with a negatively charged surface patch formed by Asp155, Glu156, Glu157, Asp159, Asp162 and Glu167 of OTUB2 (Fig. 1E). The formation of the complex buries 9% (1030 Å^2^) and 21% (1100 Å^2^) surface areas of OTUB2 and Ub, respectively, similar to the 10% (870Å^2^ ) and 22% (1020Å^2^ ) in the OTU1-Ub complex [20], and 11% (930Å^2^ ) and 23% (1080Å^2^ ) in vOTU-Ub complex.

**Figure 2:**
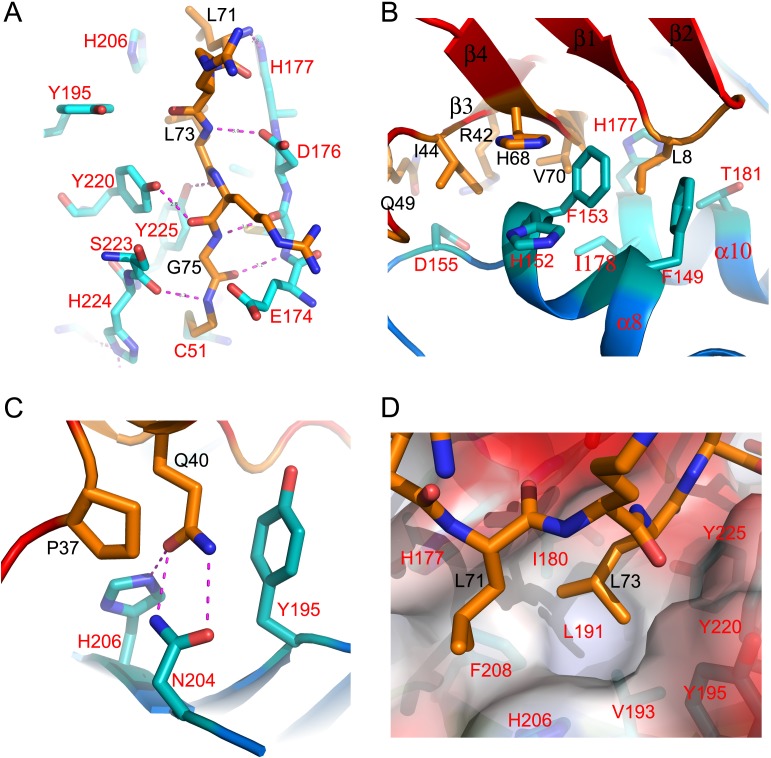
Key contact areas between OTUB2 and Ub. (**A**) Details of interactions between C-terminal tail of Ub and OTUB2. (**B**) A large contact area formed between the β-sheet of Ub and α8 and α10 helices of OTUB2. (**C**) Gln40 of Ub is fully buried in the complex interface, making stacking interactions with Tyr195 and triple hydrogen bonds to Asn204 and His206 of OTUB2. (**D**) Leu73 nests in a hydrophobic pocket formed by residues Ile180, Val193, Tyr195, His206, Phe208, Tyr220 and Tyr225 of OTUB2. The side chains of Ub are colored in cyan and those of OTUB2 in orange.

The main contact regions within the enzyme-ligand interface are formed by the C-terminal tail of Ub inserting into the cleft of OTUB2. Residues 73–75 of Ub are anchored at the bottom of the binding cleft, forming a 2-stranded antiparallel β-sheet with the α9α10 loop (residues 174–176) of OTUB2 (Fig. 2A). As expected after an SN2 type reaction of HA-UbBr2 with OTUB2, Ub Gly75 is covalently linked via—NH_2_-CH_2_-CH_2-_ to OTUB2 Cys51 (Fig. 2A). Residues from the β1β2, β3β4 and β3α3 loops of Ub make extensive interactions with α8, the α8α9 loop and α10 of OTUB2, burring several hydrophobic residues including Val70, Ile44 and Leu8 of the ligand and Phe153 of OTUB2, likely representing the main contributing area to the binding affinity (Fig. 2B). In particular, Leu8 of Ub nests in a deep hydrophobic pocket formed by residues Phe153, Phe149, Phe150, Ile178, Thr181 and His177 of OTUB2 (Fig. 2B). On the other side of the cleft, contacts are less extensive, mainly arising from α2 of Ub to β3β4, Gln40 of Ub is fully buried in the complex interface, making stacking interactions with Tyr195 and triple hydrogen bonds to Asn204 and His206 of OTUB2 (Fig. 2C). While making a network of hydrogen bond interactions to OTUB2, Leu73 from the C-terminal tail of Ub is fully buried within a hydrophobic pocket formed by residues Ile180, Val193, Tyr195, His206, Phe208, Tyr220 and Tyr225 of the enzyme (Fig. 2D).

### Comparison with other OTU-Ub structures

**Figure 3:**
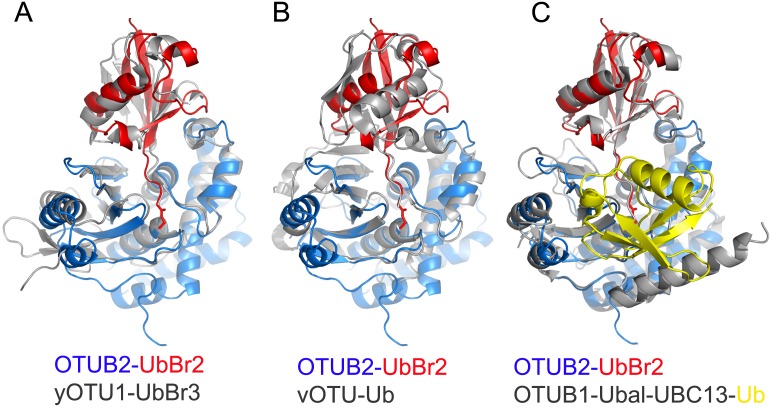
Comparison of OTUB2-Ub with other OTU-Ub complexes. Superposition of OTUB2-Ub (blue and red) with yeast OTU1-Ub (grey) [20] (**A**), vOTU-Ub (grey) [23] (**B**) and OTUB1-Ubal-UBC13-Ub (grey / yellow) [13] (**C**) complexes. The free donor Ub is shown in yellow and the UBC13 is omitted in (**C**) for clarity.

The yeast OTU1 (yOTU1)—Ub complex derived from forming a covalent bond with UbBr3 [20] shares many structural features with the human OTUB2—Ub enzyme—ligand molecule conformation (Fig. 3A). OTUB2 and yOTU1 can be imposed with 114 (out of 231) equivalent C_α_s and an rmsd of 1.4Å. In particular, the Ub ligands in both complexes have a very similar overall conformation with a modest (18°) difference in orientation to the enzyme. This is in contrast to the CCHFV derived vOTU-Ub complex [23], in which the Ub molecule is rotated by ~90° as compared to Ub in complex with OTUB2 (Fig. 3B). Interestingly, this is achieved by small differences only between the core structures of vOTU and OTUB2, represented by an rmsd of 1.7Å and 120 equivalent C_α_s (out of 156). A major hallmark of the vOTU complex is the two extra β-strands of vOTU which are involved in direct contacts with the Ub β-sheet, which in the case of OTUB2 is contacting the α8 helix. This feature appears to be unique to vOTU and may be partly responsible, in addition to the orthogonal orientation of the Ub substrate, for allowing the accommodation of both deubiquitylating and deISGylating activity [33]. Consistent with this notion, OTUB2 does not process ISG15, but Lys (K) 48/63-linked poly-Ubs and neural precursor cell expressed, developmentally downregulated 8 (NEDD8) as substrates ([5, 19], S1A Fig.). This is in contrast to OTUB1 which has a slower cleavage kinetics (S1B Fig.) and preferential specificity for Lys48-linked poly-Ub [19] [18, 19], despite a considerable structural overlap with OTUB2 (Fig. 3A and C).

### Structural differences in the N-terminal region

**Figure 4:**
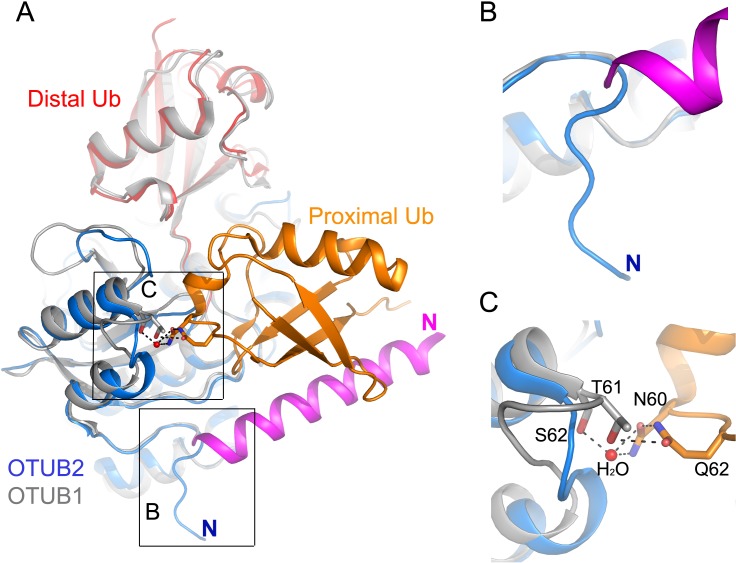
Comparison of OTUB2-Ub and OTUB1-Ubal-UBCH5B-Ub complexes. (**A**) Overlay of OTUB2-Ub (blue and red) and OTUB1-Ubal-UBCH5B-Ub (grey, grey, cyan and orange respectively) complexes. The N-terminal helix of OTUB1 is shown in magenta for clarity. Side-chains from OTUB1 and the proximal Ub are shown as sticks; black broken lines represent hydrogen bonds, and the red sphere a water molecule. (**B**) The N-terminal of OTUB2 is shorter and folded to a different direction compared to OTUB1. (**C**) Close up view of the interactions between the proximal Ub and the OTUB1 α2α3 loop which is two residue shorter in OTUB2 (α1α2 loop).

A striking difference between OTUB1 and OTUB2 is the N-terminal domain length and architecture. In the complex structure of OTUB1-Ub-UBCH5b-Ub, the proximal Ub makes extensive interactions with the N-terminal helix and α1α2 loop of OTUB1 [13–15], and the interaction with the E2 (UBC13) helps stabilizing the N-terminal α-helix [15] (Figs. 3C and 4A). The shorter N-terminal tail of OTUB2 is unstructured and oriented away from proximal ubiquitin (Fig. 4B). Notably, in the case of OTUB1, the residues Thr61 and Ser62 in the N-terminal α2α3 loop interact with proximal Ub through a hydrogen bond network with Gln62 and Asn60 (Fig. 4C). Since OTUB2 does not have the N-terminal helix and its α1α2 loop is 2 residues shorter, it is expected that the binding of proximal Ub to OTUB2 is substantially different from OTUB1.

### OTU N-termini modulate cleavage specificity towards Ub-linkages

**Figure 5:**
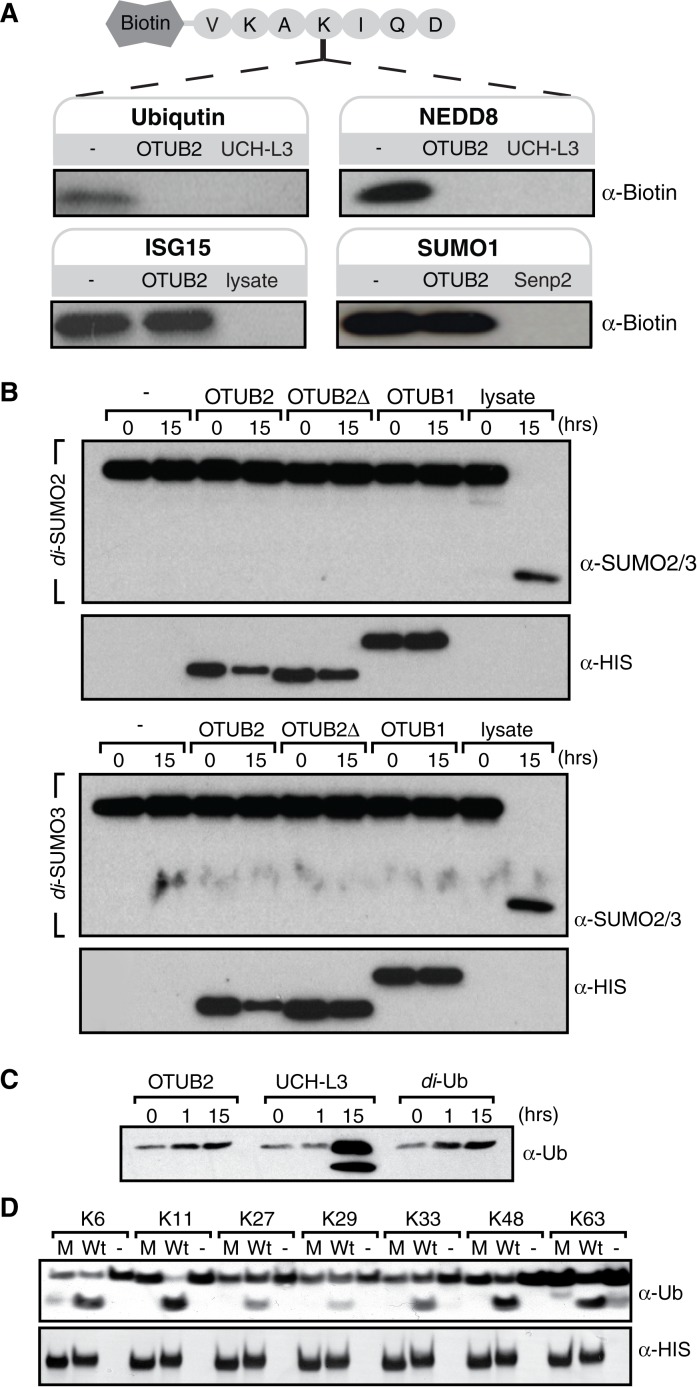
OTUB2 has a broader cleavage profile than OTUB1. (**A**) Ubiquitin (Ub), Nedd8, ISG15 and SUMO1 were conjugated to the biotinylated peptide VKAKIQD (Ub_26–32_) as described in [19] and subjected to cleavage by recombinant OTUB2, UCH-L3 or crude cell lysate (- represents untreated control), followed by SDS-PAGE separation and analysis by streptavidin-HRP immunoblotting. (**B**) di-SUMO2/3 was incubated with DMSO, OTUB2, OTUB2delta, OTUB1 or crude lysate for the indicated times, followed by SDS-PAGE separation and analysis by immunoblotting. (**C**) Linear di-Ubiquitin (di-Ub) was incubated with OTUB2, UCH-L3 or DMSO for the indicated times, followed by SDS-PAGE separation and analysis by immunoblotting. (**D**) di-Ub substrates with the linkages Lys6, 11, 27, 29, 33, 48 or 63 were incubated with either wild type (Wt) or catalytically inactive C51S mutant (M) OTUB2 for four hours, followed by SDS-PAGE and immunoblotting analysis.

**Figure 6:**
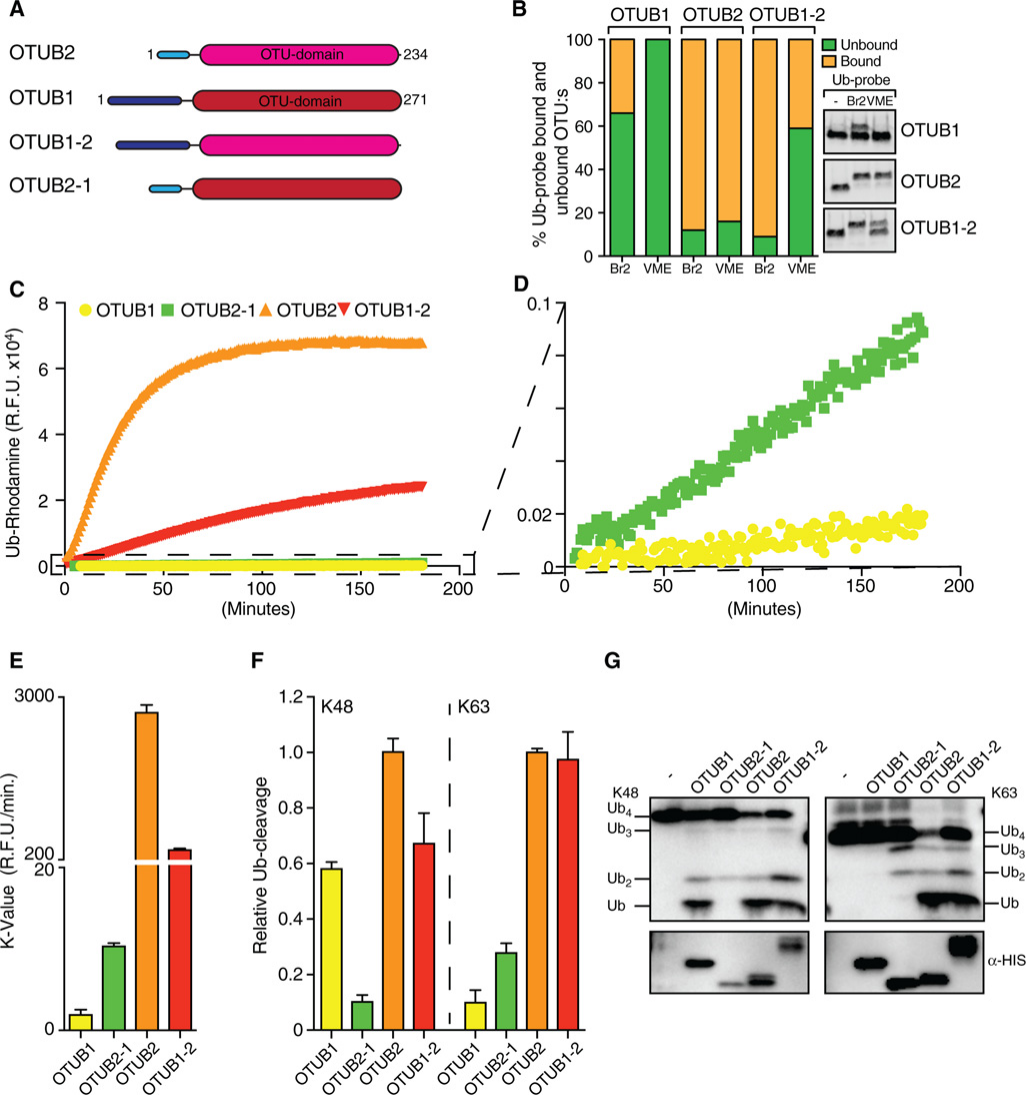
OTUB N-terminal tails modulate DUB activity and Ub chain linkage specificity. (**A**) Design of OTUB1-(N-term)-OTUB2 (Otub1–2) and OTUB2-(N-term)-OTUB1 (Otub2–1) chimera constructs and recombinant proteins (see also S2 Fig.). **(B)** Active site labeling using HA-UbBr2 (Br2) or HA-Ub-VME (VME) revealed that the OTUB1 N-terminal tail affects labeling selectivity towards the VME probe. (**C**) Ub-Rhodamine activity assay revealing the restricting effect of the N-terminal tail of OTUB1 on both, OTUB1 and OTUB2. (**D**) Magnification of the assay scale shown in (**C**) to reveal enzymatic activities of the OTUB1 (Otub1, Otub 2–1) proteins. (**E**) K-values calculated from the Ub-Rhodamine assays (**C-D**) demonstrating the restricting effect of the OTUB1 N-terminal tail. (**F-G**) Lys48 and Lys63 tetra-Ubiquitin cleavage activities are affected by OTUB1/2 N-terminal tails. For the quantitation of the relative Ub-cleavage shown in (**F**), the sum of the intensities of the bands corresponding to cleaved tetra-ubiquitin (Ub/Ub2/Ub3 observed in (**G**), upper panel) was normalized to the intensity of the band corresponding to the enzyme used (observed in the anti-his immunoblot, (**G**), bottom panel). All values in (F) are shown relative to the values observed for OTUB2 (mean of OTUB2 is set to 1), and the error bars are S.E. (n = 4).

**Figure 7:**
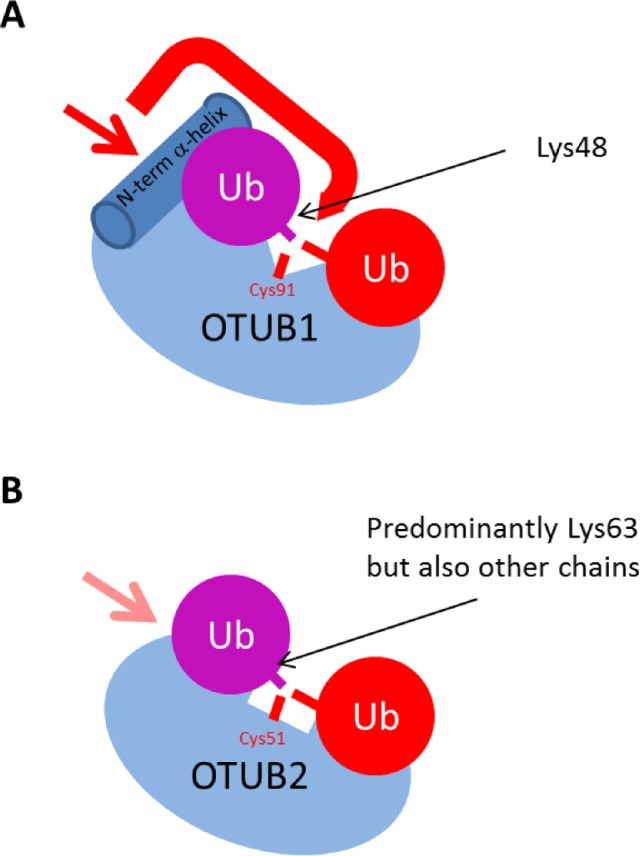
Structural features determining OTUB2’s broader cleavage specificity as compared to OTUB1. The structural models of (**A**) OTUB1 (adapted from *14*) and (**B**) OTUB2 (this study) are shown in blue, the proximal ubiquitin in purple and the distal ubiquitin in red (note that the proximal ubiquitin (purple) in (**B**) is not part of the structure). The N-terminal α-helix of OTUB1 (dark blue cylinder) that is absent in OTUB2 makes direct contact with the proximal ubiquitin and hence restricts its binding to an orientation presenting Lys48 towards the catalytic site (red arrows). This restriction is not present in OTUB2, thereby allowing a more permissive ubiquitin recognition mode.

We have searched for evidence for regulation of OTUB2 enzymatic activity. As shown previously, OTUB2 cleaved a Ub-based peptide substrate harbouring an isopeptide bond (Fig. 5A). Interestingly, we also noted cross-reactivity towards cleaving a NEDD8-based peptide substrate (Fig. 5A), although this may be a substrate-specific trait [18]. OTUB2 did not show any activity towards the ISG15-based peptide substrate, SUMO1, 2 or 3 (Fig. 5A and B) nor linear di-Ub (Fig. 5C). In contrast to OTUB1 which has exclusive specificity towards Lys48-linked chains [34], OTUB2 cleaves a broader range of di-Ub linked by naturally occurring isopeptide linkages with a preference for Lys63 di-Ub (Fig. 5D), consistent with previous studies [18]. A short C-terminal truncation (1–229; OTUB2ΔC5) did not markedly affect activity (S1A and C Fig., [5]), and no post-translational modifications within the protein were detected (data not shown). OTUB1’s strict selectivity towards cleaving Lys48-linked poly-Ub chains is in part due to its N-terminal properties [14, 15, 18]. OTUB2 has a shorter N-terminal tail and therefore might lack this feature to control for cleavage specificity. To test this hypothesis, we prepared chimeric constructs where the N-terminal tails of OTUB1 and OTUB2 were swapped to create N-term OTUB1-OTUB2 (OTUB1–2) and N-term OTUB2-OTUB1 (OTUB2–1) recombinant proteins (Fig. 6A). The OTUB1 N-terminal tails (1–83) and OTUB2 (1–43) were designated such that the OTU domain was left intact (S2 Fig.). Interestingly, active site labeling with either Br2 or VME based ubiquitin probes indicated that the OTUB1 N-terminal tail affects labeling selectivity of OTUB2 (OTUB1–2) towards the VME probe (Fig. 6B). Furthermore, OTUB2 enzymatic activity was restricted due to the presence of the OTUB1 N-terminal tail, and OTUB1 activity was enhanced in the presence of the OTUB2 N-terminal tail (OTUB2–1, Fig. 6C, D and E). Consistent with this, we observed that the presence of the OTUB1-N-terminal tail on OTUB2 (OTUB1–2) influenced its selectivity to cleave Lys63-tetra-ubiquitin chains when wild type and chimera OTUB1&2 recombinant proteins were subjected to a tetra-ubiquitin cleavage assay (Fig. 6F and G). Notably, the exclusive selectivity of OTUB1 for Lys48-linked di/tetra-ubiquitin seems to correlate with its reactivity towards the HA-UbBr2 probe with little to no reactivity towards HA-UbVME (Fig. 6B), whereas OTUB2 reacts with both Br2 and VME probes and does exhibit a more permissive cleavage profile including Lys48-, Lys63 (di/tetra-Ub)—and K6/K11 (di-Ub)-linkages (Figs. 5D, 6B and CߝG). The reason for the differential probe reactivity is not exactly understood, but clearly indicates subtle alterations within the catalytic cleft region between OTUB1 and OTUB2. In addition, structural elements other than the catalytic site must play a role as their ubiquitin chain linkage preference is also reflected by using di/tetra-ubiquitin substrates without electrophilic moieties for trapping the active site cysteine [18, 19, 35]. Crystallographic evidence suggested that the N-terminal α-helix of OTUB1 (Fig. 7, dark blue cylinder) [13, 14] that is absent in OTUB2 makes direct contact with the proximal ubiquitin and hence restricts its binding to an orientation presenting Lys48 towards the catalytic site (Figs. 4C and 7, red arrows). This restriction is not present in OTUB2, thereby potentially allowing a more permissive ubiquitin recognition mode [14, 15, 18]. OTU DUBs have been classified into different subgroups, in which OTUB1 belongs to enzymes with high selectivity for specific Ub-linkages (group I), whereas OTUB2 belongs to a set of enzymes with specificity to three of more linkage types (group III) [18]. OTUB1 and also DUBA N-terminal domains are posttranslationally modified with phosphate groups that influence their activity and/or substrate interaction [8, 36, 37]. The role of the N-terminal domain combined with some differences observed in within the catalytic cleft of OTUB1 and OTUB2 [19] could explain, at least in part, the observed differences in Ub-linkage cleavage specificity (Fig. 7). Also, it appears that other determinants, e.g. the α2α3 loop or more likely, yet to be identified interaction surfaces with the distal Ub, may be responsible for conferring chain specificity to OTUB1. Our results would be compatible with an auto-inhibitory function of the N-terminal OTUB1 helix.

Biological functions involving OTUB2 are being revealed, and structural determinations and its controlled expression pattern support a role for OTUB2 in distinct ubiquitin- dependent biological pathways. For instance, OTUB2 depletion affects the early phase of the cellular DNA damage response (DDR) [16], but also seems to control viability and insulin secretion in human beta cells [38]. In addition, OTUB2 appears to act through the inhibition of NF-κB and IFN signaling [7]. The molecular details of these processes await further investigations.

## Supporting Information

S1 Fig Comparison of wt OTUB2 and truncated OTUB2 (delta). (**A**) 150nM Lys48-, Lys63-linked tetra-ubiquitin chains were incubated at 37°C with 30 nM OTUB2 and truncated OTUB2Δ for indicated time points. The reaction was stopped by adding 3x SDS reducing sample buffer, separated by Tris-Tricine SDS-PAGE and visualized by anti-ubiquitin immunoblotting. (**B**) 50 nM of the in-house developed isopeptide TR-FRET DUB substrate (Scheme, upper panel) was incubated with recombinant OTUB2, OTUB1, OTUB1 P87G mutant and UCHL3 at the indicated concentrations (lower panel). Cleavage was measured as a ratio function of acceptor fluorescence to donor fluorescence (515/487 nm emission) as a function of time by 332 nm excitation on the Tecan Safire² Monochromator Based Plate Reader with 20 nm band pass. (**C**) Deubiquitinating activity measured by ubiquitin-AMC cleavage, a C-terminal derivatization of ubiquitin with 7-amino-4-methylcoumarin. 250 nM Ub-AMC was incubated with 100nM of OTUB2 and truncated OTUB2Δ5 (1–229). Deubiquitinating activity was determined by measuring AMC fluorescence (380/460 excitation/emission) as a function of time by fluorescence.
(PDF)

S2 Fig Sequences of OTUB1 and OTUB2 chimera constructs used in this study. The N-terminal tail of OTUB1 (UniProt accession nr Q96FW1, 1–83AA) was fused with OTUB2 (UniProt accession nr Q96DC9, 44–234AA) and the N-terminal tail of OTUB2 (1–43AA) fused to OTUB1 (84–271AA). The nucleotide and protein sequences are shown (OTUB1—yellow, OTUB2—green). The cDNA was synthesized by GeneArt (Germany) and subsequently cloned into pET28alpha vectors for bacterial expression.
(PDF)

S1 Table Data collection and refinement statistics. (PDF)
