# Supplementary figures and images for: The Human Otubain2-Ubiquitin Structure Provides Insights into the Cleavage Specificity of Poly-Ubiquitin-Linkages
Source: PLoS One. 2015 Jan 15;10(1):e0115344. doi: 10.1371/journal.pone.0115344 (PMC4295869; doi:10.1371/journal.pone.0115344)

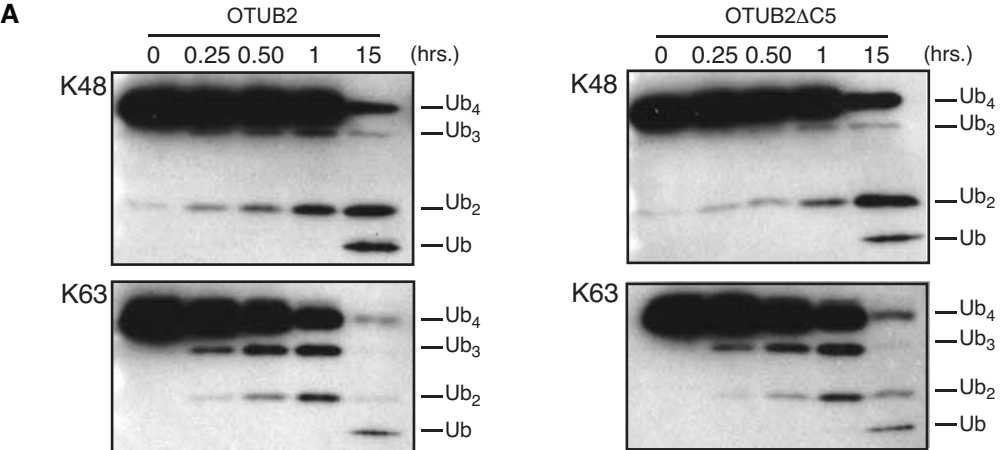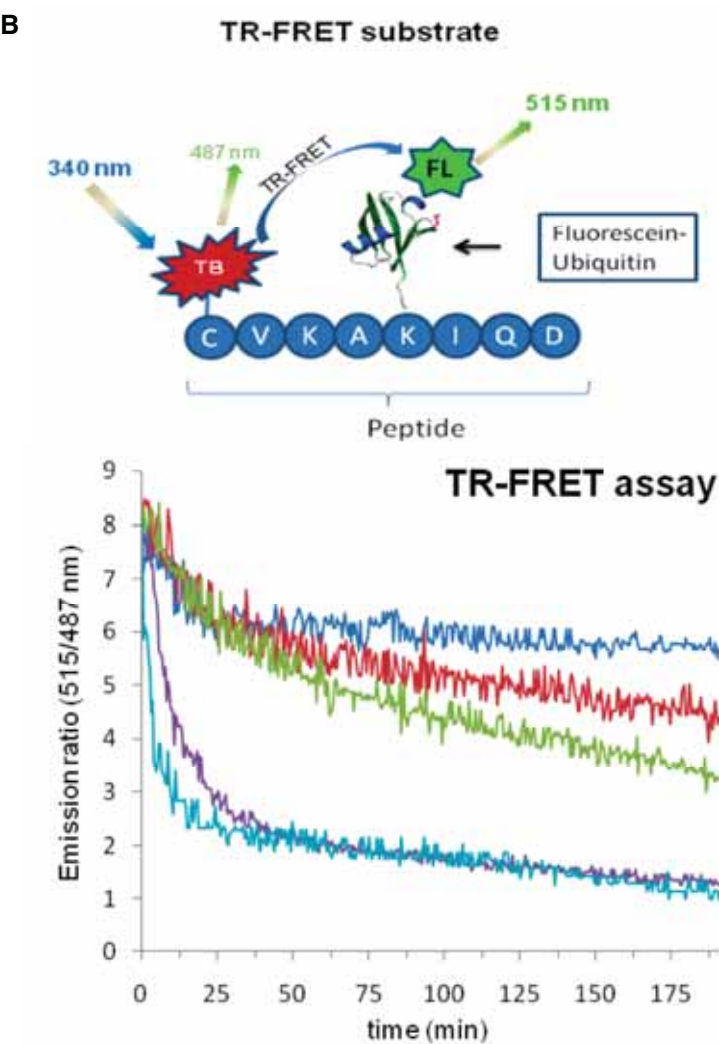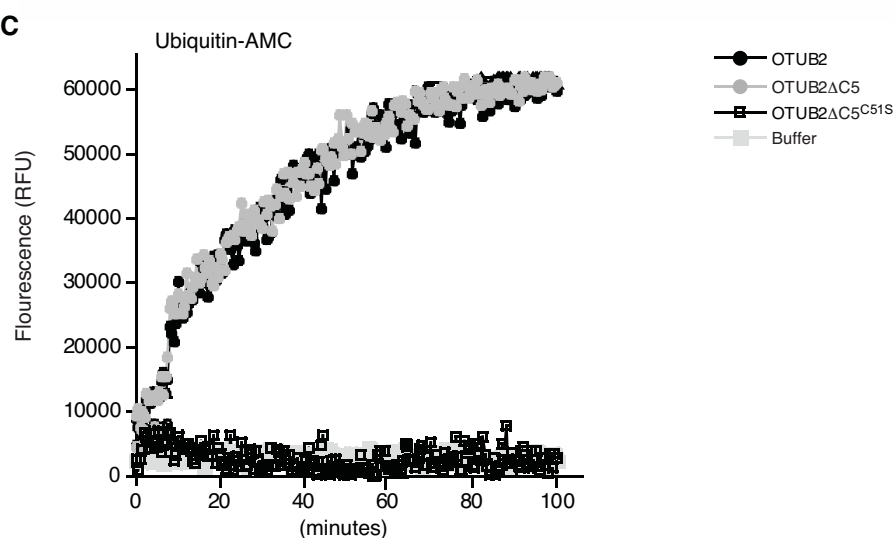

Supplement: S1 Fig — (A) 150nM Lys48-, Lys63-linked tetra-ubiquitin chains were incubated at 37°C with 30 nM OTUB2 and truncated OTUB2Δ for indicated time points. The reaction was stopped by adding 3x SDS reducing sample buffer, separated by Tris-Tricine SDS-PAGE and visualized by anti-ubiquitin immunoblotting. (B) 50 nM of the in-house developed isopeptide TR-FRET DUB substrate (Scheme, upper panel) was incubated with recombinant OTUB2, OTUB1, OTUB1 P87G mutant and UCHL3 at the indicated concentrations (lower panel). Cleavage was measured as a ratio function of acceptor fluorescence to donor fluorescence (515/487 nm emission) as a function of time by 332 nm excitation on the Tecan Safire² Monochromator Based Plate Reader with 20 nm band pass. (C) Deubiquitinating activity measured by ubiquitin-AMC cleavage, a C-terminal derivatization of ubiquitin with 7-amino-4-methylcoumarin. 250 nM Ub-AMC was incubated with 100nM of OTUB2 and truncated OTUB2Δ5 (1–229). Deubiquitinating activity was determined by measuring AMC fluorescence (380/460 excitation/emission) as a function of time by fluorescence. (PDF) [file pone.0115344.s001.pdf]
