# Supplementary material for: The Human Otubain2-Ubiquitin Structure Provides Insights into the Cleavage Specificity of Poly-Ubiquitin-Linkages
Source: PLoS One. 2015 Jan 15;10(1):e0115344. doi: 10.1371/journal.pone.0115344 (PMC4295869; doi:10.1371/journal.pone.0115344)
Supplement: S2 Fig — The N-terminal tail of OTUB1 (UniProt accession nr Q96FW1, 1–83AA) was fused with OTUB2 (UniProt accession nr Q96DC9, 44–234AA) and the N-terminal tail of OTUB2 (1–43AA) fused to OTUB1 (84–271AA). The nucleotide and protein sequences are shown (OTUB1—yellow, OTUB2—green). The cDNA was synthesized by GeneArt (Germany) and subsequently cloned into pET28alpha vectors for bacterial expression. (PDF) [file pone.0115344.s002.pdf]

FIGURE S2

OTUB1-OTUB2 (Otub1-2)

Nucleotide:

ATGGCGGCGGAGGAACCTCAGCAGCAGAAGCAGGAGCCGCTGGGCAGCGACTCCGAAGGTGTTAACTGT
CTGGCCTATGATGAAGCCATCATGGCTCAGCAGGACCGAATTCAGCAAGAGATTGCTGTGCAGAACCCT
CTGGTGTCAGAGCGGCTGGAGCTCTCGGTCCTATACAAGGAGTATGCTGAAGATGACAACATCTATCAA
CAGAAGATCAAGGACCTCCACAAAAAGTACTCGTACATCCGCAAGACCAAAGGGGATGGGAACTGCTTC
TACAGGGCCTTGGGCTATTCCTACCTGGAGTCCCTGCTGGGGAAGAGCAGGGAGATCTTCAAGTTCAAA
GAACGCGTACTGCAGACCCCAAATGACCTTCTGGCTGCTGGCTTTGAGGAGCACAAGTTCAGAAACTTC
TTCAATGCTTTTTACAGTGTGGTGGAACTGGTAGAGAAGGATGGCTCAGTGTCCAGCCTGCTGAAGGTG
TTCAACGACCAGAGTGCCTCGGACCACATCGTGCAGTTCCTGCGCCTGCTCACGTCGGCCTTCATCAGG
AACCGAGCAGACTTCTTCCGGCACTTCATTGATGAGGAGATGGACATCAAAGACTTCTGCACTCACGAA
GTAGAGCCCATGGCCACGGAGTGTGACCACATCCAGATCACGGCGTTGTCGCAGGCCCTGAGCATTGCC
CTGCAAGTGGAGTACGTGGACGAGATGGATACCGCCCTGAACCACCACGTGTTCCCTGAGGCCGCCACC
CCTTCCGTTTACCTGCTCTATAAAACATCCCACTACAACATCCTTTATGCAGCCGATAAACATTGA

Protein:

M A A E E P Q Q Q K Q E P L G S D S E G V N C L A Y D E A I **M** A Q Q D
R I Q Q E I A V Q N P L V S E R L E L S V L Y K E Y A E D D N I Y Q Q
K I K D L H K K Y S Y I R K T K G D G N C F Y R A L G Y S Y L E S L L
G K S R E I F K F K E R V L Q T P N D L L A A G F E E H K F R N F F N
A F Y S V V E L V E K D G S V S S L L K V F N D Q S A S D H I V Q F L
R L L T S A F I R N R A D F F R H F I D E E **M** D I K D F C T H E V E P
M A T E C D H I Q I T A L S Q A L S I A L Q V E Y V D E **M** D T A L N H
H V F P E A A T P S V Y L L Y K T S H Y N I L Y A A D K H

OTUB2-OTUB1 (Otub2-1

Nucleotide:

ATGAGTGAAACATCTTTCAACCTAATATCAGAAAAATGTGACATTCTATCCATTCTTCGGGACCATCCT
GAAAACAGGATTTACCGGAGGAAAATCGAGGAACTCAGCAAAAGGTTCACCGCCATCCGCAAGACCAGG
CCTGACGGCAACTGTTTCTATCGGGCTTTCGGATTCTCCCACTTGGAGGCACTGCTGGATGACAGCAAG
GAGTTGCAGCGGTTCAAGGCTGTGTCTGCCAAGAGCAAGGAAGACCTGGTGTCCCAGGGCTTCACTGAA
TTCACAATTGAGGATTTCCACAACACGTTCATGGACCTGATTGAGCAGGTGGAGAAGCAGACCTCTGTC
GCCGACCTGCTGGCCTCCTTCAATGACCAGAGCACCTCCGACTACCTTGTGGTCTACCTGCGGCTGCTC
ACCTCGGGCTACCTGCAGCGCGAGAGCAAGTTCTTCGAGCACTTCATCGAGGGTGGACGGACTGTCAAG
GAGTTCTGCCAGCAGGAGGTGGAGCCCATGTGCAAGGAGAGCGACCACATCCACATCATTGCGCTGGCC
CAGGCCCTCAGCGTGTCCATCCAGGTGGAGTACATGGACCGCGGCGAGGGCGGCACCACCAATCCGCAC
ATCTTCCCTGAGGGCTCCGAGCCCAAGGTCTACCTTCTCTACCGGCCTGGACACTACGATATCCTCTAC
AAATAG

Protein:

M S E T S F N L I S E K C D I L S I L R D H P E N R I Y R R K I E E L
S K R F T A I R K T R P D G N C F Y R A F G F S H L E A L L D D S K E
L Q R F K A V S A K S K E D L V S Q G F T E F T I E D F H N T F **M** D L
I E Q V E K Q T S V A D L L A S F N D Q S T S D Y L V V Y L R L L T S
G Y L Q R E S K F F E H F I E G G R T V K E F C Q Q E V E P **M** C K E S
D H I H I I A L A Q A L S V S I Q V E Y **M** D R G E G G T T N P H I F P
E G S E P K V Y L L Y R P G H Y D I L Y K
