# Supplementary material for: The Human Otubain2-Ubiquitin Structure Provides Insights into the Cleavage Specificity of Poly-Ubiquitin-Linkages
Source: PLoS One. 2015 Jan 15;10(1):e0115344. doi: 10.1371/journal.pone.0115344 (PMC4295869; doi:10.1371/journal.pone.0115344)
Supplement: S1 Table — (PDF) [file pone.0115344.s003.pdf]

## Table S1. Data collection and refinement statistics

| | |
|---|---|
| Data collection details: | |
| X-ray source | Diamond I04-1 |
| Wavelength ( Å ) | 0.91730 |
| Space group | $P2_12_12_1$ |
| Unit cell (Å) | *a*=54.06, *b*=76.82, *c*=198.84 |
| Resolution range (Å) | 50.0 – 2.05 (2.12-2.05) |
| Unique reflections | 52092(4955) |
| Completeness (%) | 99.1(96.1) |
| Redundancy | 9.0(6.9) |
| Average *I/σI)* | 11.0(2.5) |
| Rmerge | 0.168(0.627) |
| Refinement statistics: | |
| Resolution range (Å) | 50.0 – 2.05 |
| No. of reflections (working/test) | 49361/2686 |
| R-factor ($R_{work}/R_{free}$) | 0.212/0.269 |
| No. of atoms (protein/other atoms) | 5072/619 |
| Rms bond length deviation (Å) | 0.010 |
| Rms bond angle deviation (°) | 1.2 |
| Mean B-factor (protein/other atoms[$Å^2$]) | 36/40 |
| Ramachandran plot: | |
| Residues in preferred regions (%) | 522 (90.3) |
| Residues in allowed regions (%) | 56 (9.7) |
| Residues in disallowed regions (%) | 0 (0.0) |
